# Quantitative [^89^Zr]Zr-Trastuzumab PET and Diffusion- Weighted MRI for Characterization of Metastatic HER2-Positive Breast Cancer with PET/MRI

**DOI:** 10.2967/jnumed.124.268931

**Published:** 2025-07

**Authors:** Ameer Mansur, Jonathan E. McConathy, Erica Stringer-Reasor, Gabrielle Rocque, Katia Khoury, Noon Eltoum, Moozhan Nikpanah, Jennifer Bartels, Brian Wright, Nusrat Jahan, Denise Jeffers, Suzanne E. Lapi, Anna G. Sorace

**Affiliations:** 1 Department of Biomedical Engineering, University of Alabama at Birmingham, Birmingham, Alabama;; 2 O’Neal Comprehensive Cancer Center, University of Alabama, Birmingham, Alabama;; 3 Department of Radiology, University of Alabama, Birmingham, Alabama;; 4 Division of Hematology and Oncology, University of Alabama at Birmingham, Birmingham, Alabama; and; 5 Department of Chemistry, University of Alabama at Birmingham, Birmingham, Alabama

**Keywords:** HER2 PET/MRI, HER2-positive metastatic breast cancer, multiparametric, [^89^Zr]Zr-trastuzumab, DW-MRI

## Abstract

Current methods for evaluating HER2 expression in breast cancer are invasive and fail to capture spatial and temporal heterogeneity between primary tumors and metastases. Nuclear imaging allows for whole-body, noninvasive assessment of human epidermal growth factor receptor 2 (HER2) and can distinguish between HER2-positive and HER2-negative lesions. MRI offers superior soft-tissue contrast and quantitative metrics, such as the apparent diffusion coefficient (ADC) from diffusion-weighted (DW) imaging, providing prognostic value. The goal of this study was to present quantitative imaging metrics from [^89^Zr]Zr-trastuzumab PET and simultaneous DW-MRI to characterize HER2-positive metastatic breast cancer lesions. The secondary aim was to explore the utility of combining [^89^Zr]Zr-trastuzumab PET with DW-MRI for intratumoral habitat mapping using multiparametric [^89^Zr]Zr-trastuzumab PET/MRI to enhance characterization of lesions and assess the response to HER2-targeted therapy. **Methods:** Fifteen patients with confirmed HER2-positive breast cancer underwent simultaneous PET/MRI 5–7 d after receiving 77 ± 1.9 MBq of [^89^Zr]Zr-trastuzumab. Whole-body ADC maps were generated from DW-MRI, and regions of interest in normal and malignant tissues were delineated. Imaging metrics included SUV for PET and ADC for DW-MRI. Threshold- and clustering-based methods were applied for intratumoral characterization through multiparametric mapping. **Results:** Malignant tissues exhibited significantly higher [^89^Zr]Zr-trastuzumab uptake than did normal tissues. High uptake in the kidneys, liver, and blood pool complicated lesion identification near these tissues. ADC mapping improved lesion characterization in the brain, soft tissue, and bone. The diagnostic accuracy of [^89^Zr]Zr-trastuzumab PET alone improved when combined with ADC mapping (area under the curve, 0.59 and 0.75, respectively). Multiparametric analysis revealed intratumoral heterogeneity, identifying distinct subregions with variable tracer uptake and diffusion characteristics. **Conclusion:** Combining [^89^Zr]Zr-trastuzumab PET with DW-MRI offers a multiparametric imaging approach for characterizing HER2 expression and cellular density in HER2-positive metastatic breast cancer. Increased tracer uptake in malignant lesions and improved lesion characterization through ADC mapping highlight the potential of this combination for evaluating treatment response and tumor heterogeneity. Large-scale validation is needed to confirm these findings and support integrating [^89^Zr]Zr-trastuzumab PET and DW-MRI into clinical management for better patient outcomes.

## Visual Abstract

**Figure:**
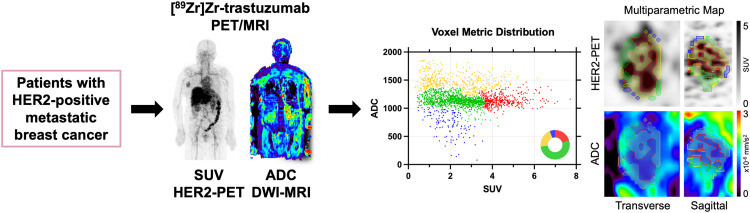

Receptor status serves as the main guiding factor for the selection of targeted therapies in breast cancer. In addition to serving as treatment targets (1–3), these receptors, such as estrogen and human epidermal growth factor receptor 2 (HER2), can be characterized though noninvasive molecular imaging (4). Current approaches for HER2 assessment, such as immunohistochemistry staining, require a tissue biopsy and are not suitable for detecting intratumoral and intertumoral spatial and temporal heterogeneity of receptor expression, which has shown value as a biomarker for response in breast tumors (5*,*^6^). This variation leads to inconsistent classification of receptor status and has the potential for suboptimal patient stratification for HER2-targeted therapies and prognosis assessment (7*,*^8^). Such discrepancies highlight the need for improved strategies in assessing HER2 status throughout the entire metastatic tumor burden that accurately assess for heterogeneous expression to optimize treatment selection for patients with metastatic HER2-positive breast cancer.

Nuclear imaging that targets HER2 expression using [^89^Zr]Zr-trastuzumab PET is a noninvasive method for addressing many of the challenges associated with traditional histology-based techniques (4). [^89^Zr]Zr-trastuzumab PET has shown to effectively distinguish between HER2-positive and HER2-negative lesions (9), detect HER2-positive metastases in patients with HER2-negative breast cancer, and exhibit increased uptake with higher immunohistochemistry scores (10–12). A recent landmark study demonstrated good diagnostic accuracy of [^89^Zr]Zr-trastuzumab PET in 200 patients with metastatic breast cancer (12). In conjunction with [^18^F]FDG PET, another study demonstrated [^89^Zr]Zr-trastuzumab PET’s capabilities to predict responders to trastuzumab emtansine therapy (13). These results are encouraging and suggest that PET can assess HER2 status throughout the tumor burden and potentially guide treatment when standard work-up is equivocal (14).

Whole-body PET is paired with a complementary anatomic imaging modality, in most cases CT. However, certain cancers (i.e., breast, brain, and prostate) benefit tremendously from the addition of the soft-tissue contrast derived from MRI. In addition to providing high-resolution soft-tissue contrast, advanced methods such as diffusion-weighted MRI (DW-MRI) have been recognized as promising imaging techniques for predicting cancer treatment response. The apparent diffusion coefficient (ADC), derived from DW-MRI, can evaluate intratumoral cellularity, which can be longitudinally monitored during anticancer treatments (15–17). Importantly, ADC has been shown to be predictive of therapeutic response in breast cancer before downstream changes in tumor size (16*,*^18^–^20^). In this study, we present quantitative imaging metrics of [^89^Zr]Zr-trastuzumab PET and simultaneous quantitative DW-MRI to characterize metastatic lesions in patients with biopsy-proven HER2-positive breast cancer. We also define standards for quantitative values across organ systems, which are critical for evaluating uptake in metastatic lesions. Additionally, we explore the utility of combining PET with DW-MRI for intratumoral habitat mapping with multiparametric [^89^Zr]Zr-trastuzumab PET/MRI to better biologically characterize lesions and lesion response to HER2 therapies. The combination of quantitative DW-MRI and [^89^Zr]Zr-trastuzumab PET has the potential to offer a clinically superior approach for understanding tumor heterogeneity in patients with metastatic HER2-positive breast cancer.

## MATERIALS AND METHODS

### Patient Population

This study (NCT03321045) was conducted as an interventional, open-label, phase 1, diagnostic imaging trial and approved by the institutional review board of the University of Alabama at Birmingham (IRB-170220004). All eligible patients gave written informed consent before participation. A flowchart of the enrolled patients is provided in Supplemental Figure 1 (supplemental materials are available at http://jnm.snmjournals.org). Fifteen patients (median age, 63 y; range, 40–80 y) diagnosed with locally advanced or metastatic HER2-positive breast cancer were enrolled between 2018 and 2024. The inclusion criteria were biopsy-proven HER2-positive status of the primary or metastatic lesion as determined by pathologic evaluation and systemic therapy that included trastuzumab or other HER2-targeted therapies, specifically trastuzumab emtansine or tucatinib. A summary of the systemic therapies patients were receiving at the time of imaging is provided in Supplemental Table 1.

Patient demographics and clinical characteristics were obtained from electronic medical records and are presented in Table 1. The presence of metastases was determined by positive findings from standard-of-care imaging, including contrast-enhanced CT, contrast-enhanced MRI, [^18^F]FDG PET, or bone scintigraphy. Five patients (33%) enrolled had regional metastases, and 10 patients (67%) had distant metastatic disease. Forty-nine cumulative lesions were identified and further filtered to 42 to exclude lesions not exceeding 1 cm in diameter. Per-lesion response was evaluated at 3 and 6 mo using follow-up anatomic imaging or pathologic assessment in accordance with RECIST 1.1 criteria. Imaging obtained closest to the time of [^89^Zr]Zr-trastuzumab PET/MRI served as the baseline for response assessment. Lesions were categorized as responding (≥30% reduction in diameter [partial response] or complete disappearance), stable (not meeting criteria for responding or progressing), or progressing (≥20% increase in diameter). For analysis, responding and stable lesions were classified as responding (favorable outcome), whereas progressing lesions were classified as nonresponding.

**TABLE 1.** Patient Characteristics (*n* = 15)

Characteristic	Value
Age (y)	63 (40–80)
White	12 (80)
Black	2 (13)
Asian	1 (7)
Disease classification	
Locally advanced	5 (33)
Metastatic	10 (67)
Total number of lesions	49
Lesions ≥ 1 cm	42
Brain	3 (7)
Breast	5 (12)
Bone	26 (62)
Soft tissue	6 (14)
Liver	2 (5)
Treatment response	
Responding disease	11 (26)
Stable disease	18 (43)
Progressing disease	13 (31)
Initial HER2 status (IHC score)	
1+	6 (40)
1 + and 2+	1 (7)
2 + and 3+	1 (7)
3+	7 (46)
Currently receiving trastuzumab therapy	
Yes	14 (93)
No	1 (7)
Pathology of primary disease	
High-grade IDC	1 (7)
IDC (including IDC with DCIS)	10 (66)
High-grade DCIS	1 (7)
DCIS	1 (7)
ILC	2 (13)

IHC = immunohistochemistry; IDC = invasive ductal carcinoma; DCIS = ductal carcinoma in situ.

Qualitative data expressed as number followed by percentage in parentheses; continuous data expressed as median followed by range in parentheses.

### [^89^Zr]Zr-Trastuzumab PET/MRI and Quantification

[^89^Zr]Zr-trastuzumab was prepared at the University of Alabama at Birmingham Cyclotron Facility using previously established methods (21). For patients not receiving trastuzumab as part of their treatment regimen, a 50-mg cold predose of trastuzumab was administered before imaging. Subsequently, each patient received 10 mg of [^89^Zr]Zr-trastuzumab, with a total mean activity of 77 ± 1.9 MBq. Patients were monitored for any adverse reactions throughout the injection, after injection, and during imaging. No adverse reactions related to the study were observed. After an uptake period of 5–7 d, simultaneous whole-body (skull vertex to thighs) [^89^Zr]Zr-trastuzumab PET/MRI was acquired for less than 90 min (Signa PET/MR; GE HealthCare) with patients positioned supine. MRI protocol included T1-weighted (liver acquisition with volume acceleration; repetition time/echo time = 4.68/1.99) and diffusion sequences with 3 *b* values (50, 500, and 800). Manufacturer MR-based attenuation corrected was used for attenuation correction.

Regions of interest (ROI) were delineated within the brain, breast, bone, kidneys, liver, lungs, and muscle on the whole-body, T1-weighted MRI in MIM (MIM Software). Tumor ROIs were manually segmented on the whole-body MRI referenced from standard-of-care imaging. As the [^89^Zr]Zr-trastuzumab PET images were coregistered to the T1-weighted MRI, these ROIs were applied to the PET data to extract SUVs (SUV_mean_, SUV_median_, and SUV_max_). Ratios of SUV_mean_ and SUV_max_ of lesions to contralateral normal tissue were calculated to provide tumor-to-background ratios (TBR_mean_ and TBR_max_). Additionally, SUV_max_ of normal breast tissue was evaluated. The ADC was extracted from DW-MRI using the standard equation:

$$\text{ADC}\;=\;–\text{ln}\left(S/S_{0}\right)/\left(b–b_{0}\right).$$ Eq. 1

Gaussian filtering was applied solely for visual representation of [^89^Zr]Zr-trastuzumab PET only.

### Multiparametric Assessment

To evaluate the predictive value of quantitative-imaging metrics across the full dataset, a logistic regression analysis was performed using single and combination metrics for all lesions. Metastatic lesions greater than 5 mL in volume on T1-weighted MRI were evaluated with multiparametric voxelwise maps incorporating coregistered [^89^Zr]Zr-trastuzumab SUV and ADC metrics. ADC maps were resampled to the resolution of the [^89^Zr]Zr-trastuzumab PET scans. Two approaches were used to assess heterogeneity through multiparametric analysis on a voxelwise basis. In the 2-step habitat map generation, thresholding based on median signal intensities from each of the SUV and ADC maps (derived separately) was performed to classify voxels independently into high- and low-intensity regions for each modality. These classifications were then combined via Boolean operations to yield 4 distinct intratumoral habitats: HighHER2–HighADC, HighHER2–LowADC, LowHER2–HighADC, and LowHER2–LowADC (22). The second method, referred to as a “1-step” approach (23), used hierarchical agglomerative clustering to group voxels based on both ADC and SUV into clusters that share similar dual-intensity profiles on a per lesion basis (23*,*^24^). Qualitative comparisons of the average SUV and ADC intensities of the multiparametric regions were performed.

For brain metastases, additional MRI sequences, including T1-weighted, T_1_ plus contrast, T2-weighted, fluid-attenuated inversion recovery (FLAIR), and DW imaging, were used to extract physiologically relevant imaging-derived ROIs (Supplemental Fig. 2). An enhanced ROI was automatically identified using T1-weighted images before and after contrast. Edema regions were identified on T2-weighted and FLAIR images. DW imaging sequences were used to identify regions of restricted diffusion within T2/FLAIR hyperintensity edema (25).

### Statistical Analysis

Descriptive statistics were calculated for demographic and clinical characteristics and presented as medians and ranges for continuous variables and frequencies and percentages for categoric variables. Statistical comparisons between groups were evaluated nonparametrically with the Mann–Whitney *U* test. Lesion segmentation and metric evaluation were performed in MIM and complemented with custom Python (Python Software Foundation) and MATLAB (MathWorks) scripts (available on request). Logistic regression modeling was conducted to assess the classification capabilities of [^89^Zr]Zr-trastuzumab uptake and ADC values in distinguishing responding and nonresponding lesions. Classification accuracy between different metrics was evaluated via the area under the receiver operating characteristic curve (AUC). All statistical comparisons were performed in Prism 10 (GraphPad).

## RESULTS

### Noninvasive Assessment of Whole-Body HER2 Biodistribution

**FIGURE 1.**
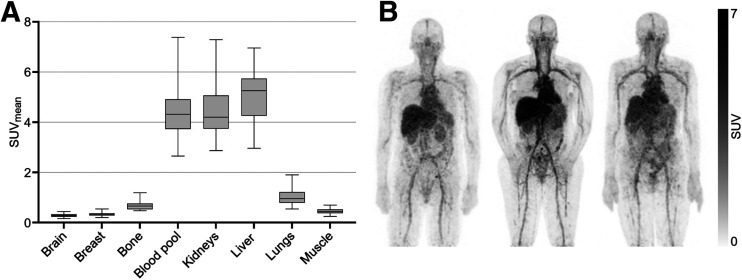
Physiologic uptake of [^89^Zr]Zr-trastuzumab across various tissues, presented as SUV_mean_ ± SD (*n* = 12–15 per tissue). (A) Differential uptake of [^89^Zr]Zr-trastuzumab in normal tissue was observed. (B) Representative whole-body [^89^Zr]Zr-trastuzumab PET maximum-intensity projection images of 3 patients, 5–7 d after administration, display heterogenous accumulation of tracer in various organs.

Use of whole-body PET acquisition allowed for evaluation of [^89^Zr]Zr-trastuzumab biodistribution throughout the entire body. Notably, minimal uptake of [^89^Zr]Zr-trastuzumab was observed in normal brain, breast, bone, lungs, and muscle tissues, with SUV_mean_ values of 0.3 ± 0.08, 0.3 ± 0.09, 0.7 ± 0.2, 1.1 ± 0.33, and 0.5 ± 0.12, respectively. However, the blood pool, kidneys, and liver demonstrated increased uptake, with an SUV_mean_ of 4.6 ± 1.0, 4.6 ± 1.1, and 5.1 ± 1.1, respectively. The stability of uptake in these key organs across the various circulation days, measured as SUV_mean_ (*P* = 0.628) and SUV_max_ (*P* = 0.828), indicates that the variation in circulation day did not affect quantification (Supplemental Fig. 3). A graphical representation of these data is presented in Figure 1A and in representative whole-body [^89^Zr]Zr-trastuzumab PET maximum-intensity projection images (Fig. 1B). The trends and differential uptake patterns were consistent across other SUV metrics and align closely with values reported in the literature (12).

### Increased [^89^Zr]Zr-Trastuzumab Uptake in Malignant Tissues

**FIGURE 2.**
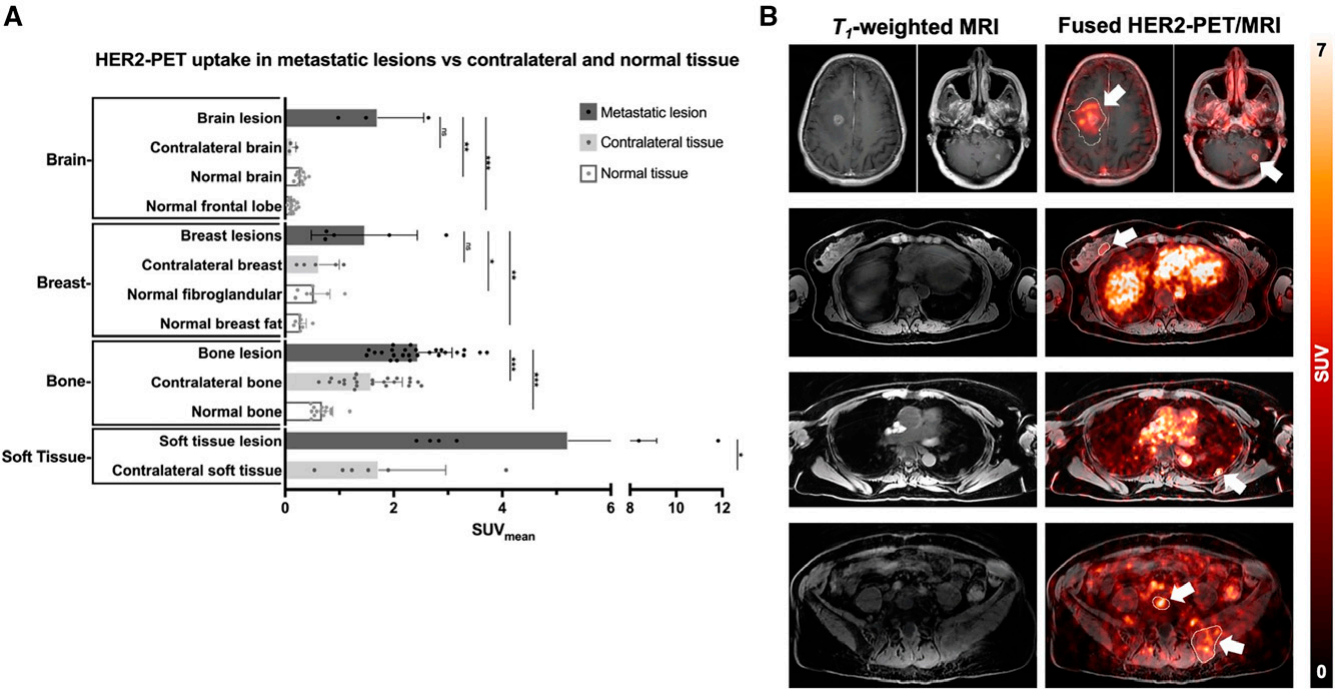
(A) Bar plots demonstrating overall increased [^89^Zr]Zr-trastuzumab uptake observed in metastatic lesions compared with contralateral and normal organ uptake. (B) Representative T1-weighted MRI and fused [^89^Zr]Zr-trastuzumab PET/MRI with primary or metastatic lesions outlined in white.

Forty-two lesions (≥ 1 cm) were identified by standard-of-care imaging, located in the brain (3 [7%]), breasts (5 [12%]), bone (26 [62%]), soft tissue (6 [14%]), and liver (2 [5%]). Overall, increased [^89^Zr]Zr-trastuzumab uptake was found in standard-of-care–confirmed malignant lesions compared with uptake in contralateral ROIs and with normal organs (Fig. 2A). Although the small sample size restricted broader statistical comparisons, uptake in the 3 contrast-enhanced brain metastases was significantly greater than that of normal brain tissue (SUV_mean_, 1.7 ± 0.85 vs. 0.14 ± 0.06, respectively; *P* = 0.004). Significant increases in uptake were also demonstrated in breast lesions compared with normal fibroglandular tissue (*P* = 0.048) and normal breast fat (*P* = 0.004). Bone lesions demonstrated significantly increased uptake compared with contralateral regions and normal bone in both paired and unpaired evaluations (*P* = 0.0001). Soft-tissue metastases also displayed a significant increase in SUV compared with contralaterally segmented regions (*P* = 0.0258). No threshold for [^89^Zr]Zr-trastuzumab PET uptake (SUV_mean_, SUV_median_, SUV_max_) differentiated responders from nonresponders, as illustrated in Supplemental Figure 4. Figure 2B presents images from paired T1-weighted MRI and fused [^89^Zr]Zr-trastuzumab PET/MRI of primary breast, breast-to-brain, breast-to-soft-tissue, and breast-to-bone metastases. SUV metrics varied widely across lesions, demonstrating heterogeneous uptake. An exploratory analysis was performed to assess the potential effect of circulating trastuzumab on [^89^Zr]Zr-trastuzumab uptake. No significant relationship was observed between the time from last therapeutic trastuzumab infusion and tumor SUV_max_ (*P* > 0.05; Supplemental Fig. 5), suggesting minimal interference from the therapeutic antibody on tracer binding.

### Utility of Simultaneous PET/MRI

**FIGURE 3.**
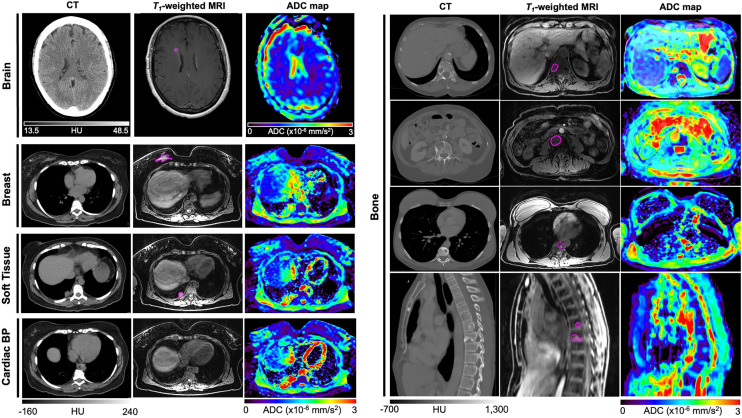
Transverse and sagittal CT, T1-weighted anatomic and corresponding ADC maps of various breast cancer metastatic lesions, outlined in magenta. Decreased ADC signal observed within enhanced regions of brain lesions is indicative of cellularly dense tissue, surrounded by regions of increased ADC, characteristic of edema. MRI and ADC allowed for clear delineation of tumors in breast and soft-tissue metastases. ADC maps allowed for accurate placement of cardiac blood pool (BP) ROI and delineation of osseous lesions borders.

MRI may provide enhanced visualization and characterization of lesions compared with conventional CT imaging, providing complementary information to PET. MRI is better than CT in delineating brain lesions and associated edema. MRI also provided tissue characterization that is not possible with CT, including incorporation of ADC mapping, which facilitates the differentiation of tissue densities. Moreover, MRI has proven invaluable not only in differentiating between fat and fibroglandular tissue within the breasts but also in allowing precise delineation of lesion borders and assessing cellular density. Additionally, the combination of MRI and ADC mapping allows for visualization of soft-tissue lesions and heterogenous cellular density, as evidenced by the images of the juxtapulmonary lesion (Fig. 3). ADC maps were also used for placement of a cardiac blood-pool ROI because a distinct spatial separation of the ventricle muscle was observed. A limitation of the chosen MRI protocol was its inability to completely visualize metastatic bone lesions, a trade-off made to minimize patient time on the table. Nevertheless, this limitation was addressed by ADC mapping, which highlighted these lesions through elevated ADC values relative to surrounding bone tissue (Fig. 3).

### Predicting Response to HER2-Targeted Therapy

As patients were enrolled during various treatment time points, we distinguished responding from nonresponding tumors as a secondary assessment. Single HER2-imaging metrics, such as SUV_mean_, SUV_median_, and SUV_max_, did not distinguish responders from nonresponders in this heterogeneous patient population (*P* = 0.5770, 0.5656, 0.7911, respectively) (Table 2). Linear correlation of the compiled metrics showed no correlation between ADC_mean_, TBR_mean_, and TBR_max_ compared with the other SUV metrics (Supplemental Fig. 6).

**TABLE 2.** Comparison of HER2-Imaging Metrics

Metric	Responders	Nonresponders	*P*
SUV_mean_	2.6 ± 1.4	2.9 ± 2.7	0.7087
SUV_median_	2.4 ± 1.4	2.6 ± 2.4	0.7589
SUV_max_	6.5 ± 3.6	7.0 ± 6.9	0.4878
ADC_mean_	1.5 ± 0.5	1.3 ± 0.5	0.1386
Contralateral-derived			
TBR_mean_	2.1 ± 1.5	5.0 ± 6.2	0.4962
TBR_max_	6.6 ± 8.5	13.8 ± 21.4	0.6215
SUV_max_/normal breast	18.5 ± 13.1	20.5 ± 24.8	0.7186
ADC-derived			
SUV_mean_ of ADC_low_ region	2.4 ± 1.4	3.1 ± 2.8	0.6310
SUV_mean_ of ADC_high_ region	2.6 ± 1.6	2.8 ± 2.5	0.6215

Data are expressed as mean ± SD.

**FIGURE 4.**
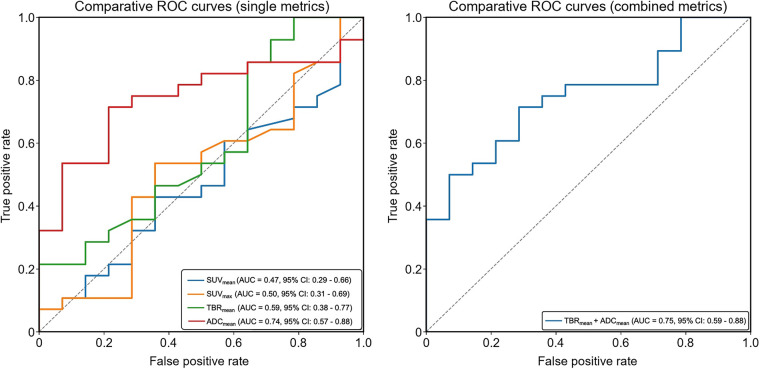
Receiver operating characteristic (ROC) curves demonstrating predictive performance of [^89^Zr]Zr-trastuzumab PET/MRI metrics for response to HER2-targeted therapy. ADC_mean_ showed highest individual predictive value (AUC, 0.74), whereas SUV_mean_ and SUV_max_ alone were not predictive. TBR_mean_ yielded modest improvement over raw SUV metrics, and its combination with ADC_mean_ produced slight increase in AUC (AUC, 0.75); however, overlapping 95% CIs suggest this was not statistically significant.

Logistic regression analysis revealed that ADC_mean_ demonstrated the strongest predictive performance for response to HER2-targeted therapy, with an AUC of 0.74 (95% CI, 0.57–0.88). In contrast, SUV-based metrics alone showed limited predictive value, with SUV_mean_ and SUV_max_ having AUCs of 0.47 (95% CI, 0.29–0.66) and 0.50 (95% CI, 0.31–0.69), respectively. Normalized uptake, defined as TBR_mean_, resulted in a modest improvement (AUC, 0.59; 95% CI, 0.38–0.77), though the CI overlapped with 0.5. When TBR_mean_ was combined with ADC_mean_, the AUC increased slightly to 0.75 (95% CI, 0.59–0.88); however, the CIs suggest that the improvement was not significantly better than ADC_mean_ alone but was better than the SUV metrics (Fig. 4).

### Tissue Characterization Through Multiparametric [^89^Zr]Zr-Trastuzumab PET and DW-MRI

**FIGURE 5.**
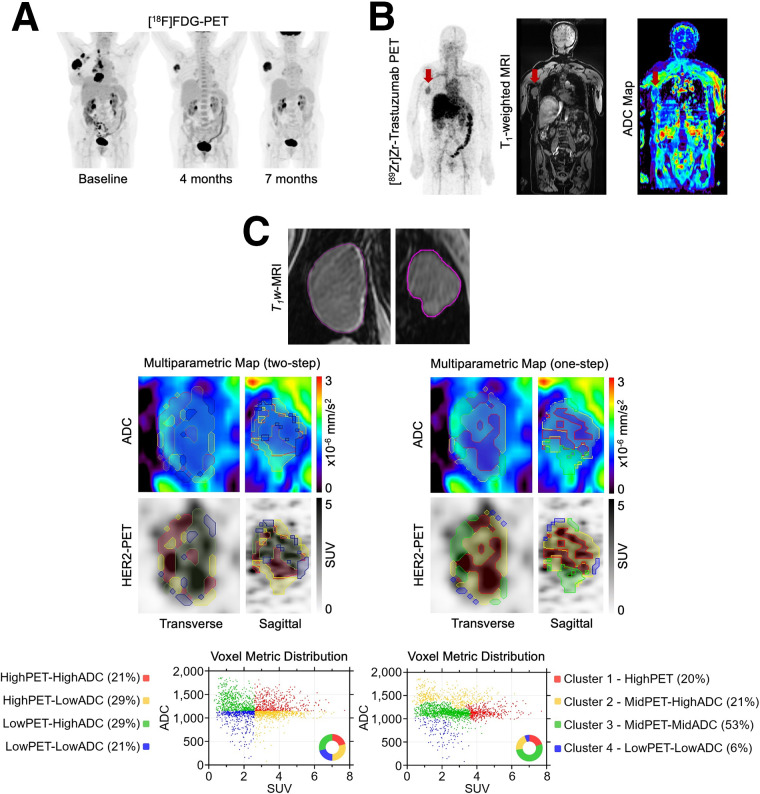
(A) Representative longitudinal [^18^F]FDG PET maximum-intensity scans showed decreased tumor burden, followed by progression. (B) Representative [^89^Zr]Zr-trastuzumab PET/MR images are shown. (C) Two multiparametric assessment techniques, with contours marking voxel classifications. Median-based thresholding (2-step method) demonstrates even split between regions based on metric intensity, with limited spatial coherence. In contrast, clustering (1-step method) identified distinct intratumoral clusters, reflected in voxel metric distribution.

Two multiparametric approaches were evaluated for tissue characterization in a large, nonresponding, metastatic axillary lymph node from a patient with HER2-positive breast cancer. After 12 treatment cycles of trastuzumab, pertuzumab, and docetaxel, [^18^F]FDG PET images (Fig. 5A) showed resolution of all lesions except for 1 axillary lymph node. Clinical follow-up after 36 cycles identified this node as unresponsive to HER2-targeted therapy, necessitating resection, and ultimately HER2-negative. Five months into therapy (after baseline), the patient was enrolled and imaged with [^89^Zr]Zr-trastuzumab PET/MRI (Fig. 5B). Heterogenous [^89^Zr]Zr-trastuzumab uptake and ADC values were observed intratumorally.

The segmentations from both methods are displayed in Figure 5C, showing improved spatial colocalization and coherence through the 1-step (clustering) method compared with 2-step median-based thresholding. Scatter plots of SUV versus ADC for the voxels and associated classes demonstrated the ability of both techniques to identify distinct regions of varying underlying biology, with the 1-step method providing more distinct identification of intratumoral subregions. Associations between the identified clusters and physiologic regions were also noted.

Cluster 1 demonstrated high HER2-expressing, cellularly dense regions, typically representative of aggressive HER2-positive regions. Cluster 2 showed a range of HER2 expression in regions with lower cellular density, indicating potential regions of response and decreasing HER2 dependence. Comprising 53% of the entire tumor, cluster 3 demonstrated low HER2 uptake and increased cellular density (decreased ADC), suggesting a large subtumoral population of low HER2-expressing regions that are actively proliferating and less likely to respond to HER2-targeted treatment. Given the limited sample size and reference standards, our focus was on demonstrating the technique’s characterization capability rather than drawing definitive conclusions about tumor characteristics.

**FIGURE 6.**
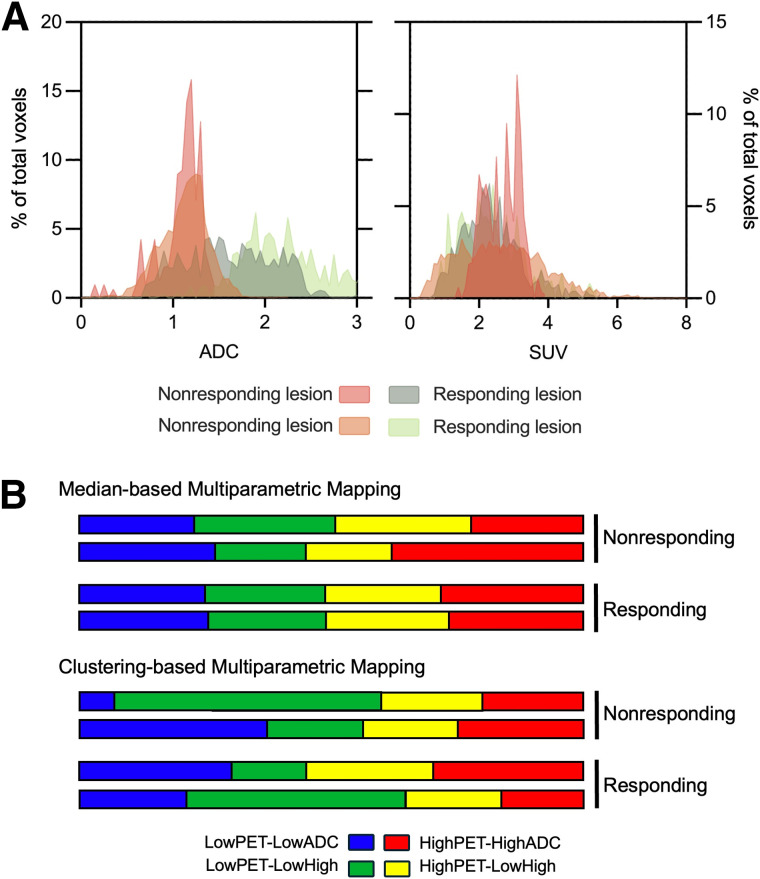
Quantitative multiparametric evaluations of breast-to-brain metastases using [^89^Zr]Zr-trastuzumab PET/ADC compared with physiologic habitats: edema with restricted diffusion, tumor enhanced core, vasogenic edema, and residual. (A) Representative transverse [^89^Zr]Zr-trastuzumab PET, anatomic T1-weighted, ADC, and T2/FLAIR sections of brain lesion are displayed, with overlay of multiparametric maps on using median intensity separation (2-step) and clustering (1-step) of ADC and SUV values (B). (C) Bar plots of SUV_mean_ and ADC demonstrated improved separation between 1-step, 2-step, and physiologic regions. Voxelwise ADC vs. SUV plots illustrate overlap of dual-intensity voxels.

As the brain offers a unique microenvironment for tumor growth, the multiparametric technique was performed to assess tissue heterogeneity in a large brain metastasis. Voxel classification within the ROI was performed by leveraging a multimodal approach that incorporated standard-of-care imaging. This stratified the lesion into distinct physiologic habitats, delineating enhanced tumor regions, vasogenic edema, and edema with restricted diffusion. [^89^Zr]Zr-trastuzumab PET images demonstrated heterogenous uptake that extended beyond the T1-enhanced region without encompassing the entire enhanced T2/FLAIR or increased ADC region (Fig. 6A). As expected, the low ADC region within the area of interest corresponds to the high cellular density of the enhanced region. To identify the characterization capabilities of both modalities combined, 2 multiparametric strategies were evaluated. The resulting maps are overlayed on the anatomic T1-weighted MRI in Figure 6A. Figure 6B presents a quantitative assessment of these methods, with bar graphs of the mean ADCs and SUV intensities separated by similar profiles.

Using nonspatial [^89^Zr]Zr-trastuzumab PET and ADC data alone, both techniques identified the enhanced core with intensities resembling physiologic segmentations. The signatures in the other regions demonstrated large variance compared with matched physiologic segmentations, indicating that both multiparametric methods identified distinct subregions to those identified physiologically by MRI alone. Scatterplots of ADC versus SUV demonstrate the voxel wise overlap of similar dual-intensity voxels and their classification based on each method (Fig. 6C). Although the 2-step method cleanly isolated the voxel groups, it may be less robust with more homogenous multiparametric data compared with the 1-step method, as it is more challenging to separate the distributed data.

**FIGURE 7.**
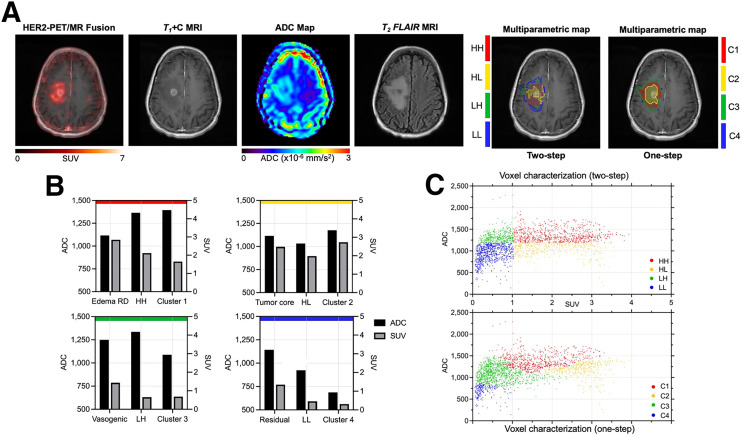
Qualitative analysis of large metastatic lesions revealed distinct patterns in ADC and SUV distributions. (A) SUV was more homogenous in responding tumors. (B and C) Nonresponding lesions showed homogenous ADC distributions skewed toward lower values, indicating increased cellular density, whereas responding lesions had more heterogeneous ADC distributions. HH = high PET/high ADC; HL = high PET/low ADC; LH = low PET/high ADC; LL = low PET/low ADC; RD = restricted diffusion.

Next, multiparametric techniques were applied to 4 lesions across 3 patients that met the volume threshold of at least 5 mL, of which 2 were classified as nonresponding tumors and 2 as responding tumors based on conventional imaging and the aforementioned criteria. Although each lesion evaluated resided in different tissue, histogram distributions of ADC demonstrated a more homogenous distribution centered toward lower ADC values in the nonresponding lesions, indicative of increased cellular density (Fig. 7A). Responding lesions demonstrated a heterogenous distribution of ADC, representing populations of higher and lower cellular densities. SUV distributions were normally distributed and centered around an SUV of 2 to 3. Although the sample size limits quantitative comparisons, qualitative assessment of the relative volumes of each habitat revealed dual-metric intratumoral heterogeneity (Fig. 7B). Two-step multiparametric mapping yielded a more even split across habitats, whereas 1-step mapping allowed for enhanced characterizations of the intratumoral subregions. Minimal trends were observed between these lesions’ intratumoral heterogeneity of metrics and treatment response. Although limited, this approach demonstrates the capabilities of PET/MRI to characterize intratumoral subregions based on their dual-metric HER2 expression and cellular density.

## DISCUSSION

This study investigated the potential of whole-body [^89^Zr]Zr-trastuzumab PET/MRI for the noninvasive assessment of whole-body HER2 expression in metastatic HER2-positive breast cancer. Although [^89^Zr]Zr-trastuzumab PET/CT has been studied, this is the first investigation incorporating simultaneous quantitative MRI with [^89^Zr]Zr-trastuzumab PET. Patients were enrolled between 2018 and 2024, a period of evolving systemic therapy options for HER2-positive metastatic breast cancer. All patients evaluated were clinically classified as having HER2-positive breast cancer; HER2-low disease was not present in this cohort. Key findings of this study include increased uptake of [^89^Zr]Zr-trastuzumab in malignant tissue versus contralateral and normal organs, improved diagnostic capabilities when combining SUV metrics and ADC_mean_, opportunities for PET/MRI in brain metastases, and effective characterization of intratumoral HER2 heterogeneity.

Limited data exist on [^89^Zr]Zr-trastuzumab uptake in healthy tissues. Establishing clear thresholds is crucial for accurately distinguishing between malignant and normal tissue. To address this, this study reported physiologically normal uptake in brain, breast, bone, lung, blood pool, liver, kidneys, and muscle, as well as uptake in lesions. The findings revealed uptake values in both normal and malignant tissues that were consistent with that of Dijkers et al. (26) while including additional metastatic sites of HER2-positive breast cancer. MRI offered both qualitative and quantitative utility, as the inherent soft-tissue contrast is critical for visualizing brain, breast, and soft-tissue lesions. Increased ADC enabled visualization and delineation of the osseous lesions. High uptake in the liver after [^89^Zr]Zr-trastuzumab administration. presumably related to metabolism. likely limited the characterization of liver metastases with this technique. This study confirmed the low uptake of [^89^Zr]Zr-trastuzumab in normal brain tissue and brain metastases, as well as in perimetastatic regions of edema, which, to our knowledge, has not been previously reported.

Although initial evaluations of the heterogeneity of HER2 expression in breast cancer used [^89^Zr]Zr-trastuzumab PET alone (9–11), by combining it with additional imaging, such as [^18^F]FDG PET/CT, the ZEPHIR trial demonstrated improved positive predictive values with trastuzumab emtansine compared with either modality alone (13). Though promising and supportive of multiparametric imaging, acquiring 2 PET scans presents challenges. PET/MRI allows for simultaneous 1-time acquisition of the antibody-based tracer, an anatomic reference, and quantitative MRI sequences. Although [^18^F]FDG PET/CT was used for lesion detection as part of clinical care in this study, variability in timing, acquisition, and registration precluded its inclusion in quantitative analysis. Future studies incorporating standardized [^18^F]FDG PET/CT alongside [^89^Zr]Zr-trastuzumab PET and DW-MRI should evaluate the comparative and complementary roles of metabolic, structural, and receptor-targeted imaging in metastatic breast cancer (13). Our findings suggest that the inclusion of DW-MRI metrics, such as ADC, improves the predictive performance of [^89^Zr]Zr-trastuzumab imaging by allowing for characterization of cellular density. Moreover, multiparametric antibody-based PET/MRI can characterize intratumoral subregions. This approach has the potential to guide personalized therapy and enhance the understanding of antibody-based treatment delivery through measurement of receptor expression and cellular density in a single imaging session.

A correlation between [^89^Zr]Zr-trastuzumab uptake and lesion-based response to therapy was not observed in this study, and the data did not support the existence of a clear [^89^Zr]Zr-trastuzumab PET uptake threshold predictive of treatment response for patients undergoing treatment, likely due to heterogeneity in our patient cohort. Regression analysis indicated that ADC_mean_ outperformed [^89^Zr]Zr-trastuzumab PET SUV metrics alone in predicting response to HER2-targeted therapy during treatment. Combining TBR_mean_ with ADC_mean_ resulted in a slight increase in AUC; however, overlapping CIs suggest no significant improvement. This demonstrates the need for complementary metrics before treatment, as indicated in the ZEPHIR trial, and during treatment (13). Therapy-induced alterations in tumor cellularity may account for the enhanced predictive value of ADC. One limitation of semiquantitative PET/MRI is its limited resolution, which prevents analysis of lesions smaller than 1 and 5 mL for multiparametric assessment. Although voxel-level multiparametric analysis was restricted to lesions larger than 5 mL, a logistic regression model using extracted summary statistic values was applied to all lesions. This complementary approach enabled lesion-level evaluation of the predictive value of [^89^Zr]Zr-trastuzumab PET and MRI metrics, even in smaller lesions not suitable for voxelwise assessment because of partial-volume effects. Imaging and multiparametric habitat analysis may have shown even more impact in pretreatment cohorts. Restricting imaging to pretreatment or during progression may provide better timing for multiparametric PET/MRI.

Our findings did not demonstrate a consistent relationship between [^89^Zr]Zr-trastuzumab uptake and lesion response to HER2-targeted therapy. Although ADC_mean_ showed a trend toward higher values in responding lesions (*P* = 0.1386), no statistically significant association was found. The lack of correlation between SUV_mean_ and ADC_mean_ across lesions (Supplemental Fig. 7) suggests that [^89^Zr]Zr-trastuzumab PET and DW-MRI capture distinct biologic features: molecular target expression and tissue cellularity. The lower number of lesions per patient reflects the real-world setting of patients actively receiving HER2-targeted therapy, in contrast to studies such as IMPACT-MBC, which enrolled patients with newly diagnosed metastatic disease, a population that typically has a higher disease burden. Dynamic changes in HER2 status and site-specific discordance limit the reliability of biopsy results. Although concurrent biopsies at the time of imaging would provide direct validation of HER2 status, many metastatic lesions—such as those in this study—are not amenable to sampling. We focused on the use of [^89^Zr]Zr-trastuzumab PET to assess these hard-to-biopsy lesions. Although concurrent therapies may contribute to response heterogeneity, this reflects the real-world treatment landscape, where multiagent regimens, particularly those involving various HER2-targeted agents, are the standard of care.

Building on these findings, future research should focus on validating these techniques and results in larger cohorts with more homogeneous treatment histories. The ZEPHIR study found a strong predictive value of pretreatment [^89^Zr]Zr-trastuzumab PET (particularly in combination with early [^18^F]FDG PET) in patients uniformly treated with trastuzumab emtansine and confirmed HER2-positive (immunohistochemistry score, 3+) disease. In contrast, our imaging study was conducted during treatment, introducing heterogeneity in response, regimens, HER2 classification, and timing of imaging which limited prediction capabilities. The results of the IMPACT-MBC study further support the impact of this heterogeneity, showing substantial intrapatient and interpatient HER2 expression variability across more than 5,000 lesions and discordance with biopsy results. Together, these results highlight the complementary nature of PET and MRI biomarkers, though further validation in larger, standardized cohorts is needed to define their predictive value. HER2 status in this study was based on findings from primary tumor biopsies, as sampling of metastatic lesions was not feasible in most patients. As a result, lesion-level correlation between [^89^Zr]Zr-trastuzumab PET uptake and HER2 immunohistochemistry score was not performed. The recent IMPACT-MBC study demonstrated a clear relationship between HER2 immunohistochemistry score and [^89^Zr]Zr-trastuzumab PET SUV_max_, supporting the potential of molecular imaging to differentiate HER2 expression levels, including HER2-low disease (12). Future studies including biopsies of metastatic lesions and paired imaging could assess whether MRI parameters, such as ADC, improve the classification of equivocal lesions. Standardized [^18^F]FDG PET, alongside [^89^Zr]Zr-trastuzumab PET and DW-MRI, should be used in comparative studies to evaluate or clarify the complementary roles of metabolic, structural, and receptor-targeted imaging when characterizing metastatic breast cancer. Longitudinal studies tracking [^89^Zr]Zr-trastuzumab uptake and cellular density over the course of treatment will provide valuable insights into dynamic tumor biology in response to therapy. [^89^Zr]Zr-trastuzumab PET imaging could also be expanded to populations with HER2-low disease, whose therapeutic relevance has grown in recent years. Ultimately, the continued development and clinical implementation of this multiparametric imaging approach has the potential to improve disease characterization and guide personalized treatment strategies to enhance outcomes for patients with metastatic HER2-positive breast cancer.

## CONCLUSION

This study demonstrated the potential of [^89^Zr]Zr-trastuzumab PET combined with DW-MRI as a multiparametric imaging approach in HER2-positive metastatic breast cancer. Although [^89^Zr]Zr-trastuzumab PET effectively distinguished HER2-expressing metastatic lesions from normal and contralateral tissue, PET-derived uptake metrics were not individually predictive of treatment response in patients undergoing active HER2-targeted therapy. In contrast, DW-MRI–derived ADC values, which reflect tumor cellularity, demonstrated stronger predictive performance. Combining [^89^Zr]Zr-trastuzumab PET with ADC did not significantly improve response prediction beyond ADC alone. Further large-scale validation is crucial to confirm these findings in a broader patient population. Integrating PET and MRI into the management of HER2-positive breast cancer provides valuable insights into tumor biology and treatment response and has the potential to transform patient care and significantly improve outcomes.

## DISCLOSURE

This work was supported by the O’Neal Comprehensive Cancer Center, Department of Radiology at University of Alabama at Birmingham, and an NIH P30 grant (5P30CA013148-480). No other potential conflict of interest relevant to this article was reported.

## Notes

KEY POINTS **QUESTION:** Does combining [^89^Zr]Zr-trastuzumab PET with DW-MRI improve the characterization of HER2-positive metastatic breast cancer by assessing intratumoral heterogeneity and enhancing diagnostic accuracy? **PERTINENT FINDINGS:** In this prospective study of 15 patients with HER2-positive metastatic breast cancer, simultaneous [^89^Zr]Zr-trastuzumab PET/MRI was used to characterize tumor biology. Although PET effectively identified HER2-expressing lesions, PET-derived SUV metrics alone were not predictive of treatment response. DW-MRI–derived ADC values, reflecting tumor cellularity, showed stronger predictive value. Combining PET and MRI improved diagnostic performance and enabled intratumoral characterization of HER2 expression and cellular density. **IMPLICATIONS FOR PATIENT CARE:** Integrating [^89^Zr]Zr-trastuzumab PET with DW-MRI offers a multiparametric imaging approach that may enhance lesion characterization and provide deeper biologic insight into HER2-positive metastatic disease. This could support more precise treatment planning and improved assessment of therapeutic response, though further validation is warranted.
